# Anatomic Features of the Nasal and Pharyngeal Region Do Not Influence PAP Therapy Response

**DOI:** 10.3390/ijerph20166580

**Published:** 2023-08-15

**Authors:** Christopher Seifen, Nadine Angelina Schlaier, Johannes Pordzik, Anna-Rebekka Staufenberg, Christoph Matthias, Haralampos Gouveris, Katharina Bahr-Hamm

**Affiliations:** Sleep Medicine Center, Department of Otolaryngology, Head and Neck Surgery, University Medical Center Mainz, 55131 Mainz, Germany

**Keywords:** obstructive sleep apnea, OSA, nasal obstruction, pharyngeal obstruction, positive airway pressure, PAP

## Abstract

The objective of this study was to investigate to which extent anatomic features of the nasal and pharyngeal region contribute to the severity of obstructive sleep apnea (OSA) and positive airway pressure (PAP) therapy response. Therefore, 93 patients (mean age 57.5 ± 13.0 years, mean body mass index 32.2 ± 5.80 kg/m^2^, 75 males, 18 females) diagnosed with OSA who subsequently started PAP therapy were randomly selected from the databank of a sleep laboratory of a tertiary university medical center. Patients were subdivided based on nasal anatomy (septal deviation, turbinate hyperplasia, their combination, or none of the above), pharyngeal anatomy (webbing, tonsillar hyperplasia, their combination, or none of the above), and (as a separate group) tongue base anatomy (no tongue base hyperplasia or tongue base hyperplasia). Then, polysomnographic data (e.g., arousal index, ARI; respiratory disturbance index, RDI; apnea index, AI; hypopnea index, HI; and oxygen desaturation index, ODI) of diagnostic polysomnography (PSG) and PAP therapy control PSG were collected, grouped, and evaluated. Septal deviation, turbinate hyperplasia, or their combination did not significantly affect the assessed PSG parameters or the response to PAP therapy compared with patients without nasal obstruction (*p* > 0.05 for all parameters). Accordingly, most PSG parameters and the response to PAP therapy were not significantly affected by webbing, tonsil hyperplasia, or their combination compared with patients without pharyngeal obstruction (*p* > 0.05 for RDI, AI, HI, and ODI, respectively). However, in the pharyngeal anatomy group, ARI was significantly higher in patients with tonsil hyperplasia (*p* = 0.018). Further, patients with tongue base hyperplasia showed a significantly higher HI in the diagnostic PSG (*p* = 0.025) compared with patients with normal tongue base anatomy, but tongue base anatomy did not significantly affect the response to PAP therapy (*p* > 0.05 for all parameters). The influence of anatomic features of the nasal and pharyngeal region on PAP therapy response appears to be small, and generalizability of these results requires further studies.

## 1. Introduction

Obstructive sleep apnea (OSA) is considered the most relevant form of sleep-disordered breathing (SDB), and its prevalence is increasing [1]. On an anatomical level, OSA is defined by the upper airways not being kept sufficiently open during sleep. This results in partial to complete airway obstruction, even when respiratory effort is still present [2]. Furthermore, the pathogenesis of OSA is based not only on anatomic factors (such as upper airway collapsibility) but also on potential contributions of non-anatomic factors, such as decreased responsiveness of pharyngeal muscles, decreased arousal threshold, increased loop gain, or disordered central motor output [3,4]. To assess the severity of OSA, the apnea–hypopnea index (AHI) or the respiratory disturbance index (RDI) are commonly used [5,6]. Typically, OSA significantly impairs the quality of life. The main reasons for this are snoring, sleep fragmentation, and pronounced daytime sleepiness [1]. The first-line therapy for moderate to severe sleep apnea is the usage of positive airway pressure (PAP) to keep an individual’s airway open throughout sleep [5,7]. Large epidemiologic studies have found that the prevalence of mild OSA ranged from 9 to 24% in people aged 30–60 years [8], 26 to 28% in people aged 30–70 years [9], and 17% in people aged 20–99 years [10,11]. Accordingly, the prevalence of moderate OSA ranged from 4 to 9% in people aged 30–60 years [8], from 7 to 14% in people aged 30–70 years [9], and from 2 to 7% in people aged 20–99 years [10,11]. Based on the average of prevalence estimates from these studies in predominantly white men and women with a mean body mass index (BMI) of 25–28 kg/m^2^, it has been estimated that approximately one in five adults has at least mild OSA and one in fifteen has at least moderate OSA [12]. Previous research has linked OSA to the risk of developing glaucoma [13], the risk of developing non-alcoholic hepatic steatosis [14], poorer oncologic therapy outcomes for head and neck tumors [15], the development of coronary artery disease [16], the occurrence of stroke [17], the development of arterial hypertension [18], and adverse health outcomes beyond these [19]. In summary, OSA has a significant public health impact and is estimated to affect one billion people worldwide between the ages of 30 and 69 [20]. Being overweight and particularly having an elevated BMI are considered the most important risk factors for the development of OSA [21]. In addition, anatomic features of the nasal region (e.g., septal deviation and turbinate hyperplasia), the pharyngeal region (e.g., tonsil hyperplasia and a low standing and sagging palate called “webbing”), and tongue base hyperplasia are discussed risk factors for OSA [5]. However, it remains controversial whether nasal or pharyngeal obstruction alone is related to OSA severity or even affects PAP therapy. This matter has only been studied in smaller cohorts with conflicting study results [22,23,24,25,26,27,28,29,30]. Consequently, the objective of our study was to retrospectively examine and compare polysomnographic parameters before and after the start of PAP therapy, taking baseline study population characteristics and different anatomic features of the nasal region (e.g., septal deviation and turbinate hyperplasia), the pharyngeal region (e.g., tonsil hyperplasia and webbing), and the tongue base anatomy (e.g., no tongue base hyperplasia and tongue base hyperplasia present) into consideration.

## 2. Materials and Methods

### 2.1. Design

We investigated the databank of our sleep laboratory (which is part of a tertiary university medical center) for patients who underwent first-time full-night polysomnography (PSG). A specifically trained technician performed the PSG overnight. Each PSG was analyzed and interpreted by a physician (sleep medicine specialist) based on the American Academy of Sleep Medicine (AASM) guidelines of 2007 [31]. Hypopnea was scored according to the update of 2012. A decrease of 30% in respiratory effort combined with either an arousal or a decrease of at least 3% in oxygen saturation constitutes hypopnea [32]. Patients included in this study were randomly selected from the clinical databank. 

Inclusion criteria consisted of:

- The initial diagnosis of OSA according to the International Classification of Sleep Disorders (ICSD) criteria [31] after performance of a full night inpatient PSG,
- the consent to start PAP therapy (devices used were Phönix 2 or Respironics REMstar Auto, Philips Respironics Inc., Murrysville, PA, USA; typically, with nasal masks),
- the subsequent performance of a full night inpatient PSG for PAP therapy control (usually performed three months after PAP therapy begins; adherence to PAP therapy ≥ 4 h for ≥70% of nights), and
- age of 18 years or older.

Exclusion criteria were:

- The diagnosis of a type of SDB other than OSA (e.g., periodic breathing or Cheyne–Stokes respiration),
- known history of chronic obstructive or restrictive lung disease,
- usage of alternative types of noninvasive positive airway pressure therapy (e.g., CPAP and BiPAP),
- already being under PAP therapy, and
- age under 18 years.

First, all patient characteristics were evaluated (e.g., patient age, sex, and BMI), then each medical file was evaluated for descriptions of anatomic features of the nasal and pharyngeal region from the otolaryngologic examination, which was performed and documented prior to first-time PSG. All otolaryngologic examination results were based on clinical (visual) examination consensus by a team consisting of a resident physician plus an experienced board-certified ears, nose, and throat (ENT) physician in a tertiary university medical center. Computed tomography or magnetic resonance imaging was not included to classify individual obstruction.

To determine the impact of nasal obstruction on the severity of OSA and PAP therapy response, patients were classified according to the following anatomic features:

(1) No nasal obstruction,
(2) septal deviation present,
(3) turbinate hyperplasia present, or
(4) septal deviation and turbinate hyperplasia present.

To assess the impact of pharyngeal obstruction on OSA severity and PAP therapy response, patients were divided into groups based on the following anatomic features:

(1) No pharyngeal obstruction,
(2) webbing present,
(3) tonsillar hyperplasia present (Friedman grading scale ≥ 3) [33], or
(4) webbing and tonsillar hyperplasia present.

To analyze the influence of the tongue base on the severity of OSA and PAP therapy response, the anatomy was classified as follows, and patients were then divided into groups:

(1) No tongue base hyperplasia or
(2) tongue base hyperplasia present (relaxed tongue sits above the occlusal plane of the mandibular teeth [34]).

Then, both PSG recordings of each patient (diagnostic PSG and PAP therapy control PSG—usually performed three months after PAP therapy begins) were analyzed for the following parameters (in alphabetical order):

- Apnea index (AI): number of apneic events per hour (h) of sleep,
- arousal index (ARI): number of arousals/h of sleep,
- hypopnea index (HI): number of hypopnea events/h of sleep,
- oxygen desaturation index (ODI): number of oxygen desaturation events (≥4%)/h of sleep, and
- respiratory disturbance index (RDI): number of abnormal breathing events/h of sleep (apneas + hypopneas + RERAs, where RERA (respiratory effort-related arousal) is defined as an episode characterized by an increased respiratory effort caused by upper airway airflow reduction resolved with arousal and accompanied in most cases by hypoxemia [35]).

The primary goal of our study was to determine the effect of nasal and pharyngeal anatomic features (septal deviation/turbinate hyperplasia and webbing/tonsillar hyperplasia/tongue base hyperplasia, respectively) on PAP therapy response. To do so, the following procedure was used: first, the diagnostic PSG parameters (ARI, RDI, AI, HI, ODI) were compared within the different groups of anatomic features. Then, the same procedure was followed for the PAP therapy control PSG parameters. Subsequently, the delta (Δ) for the diagnostic PSG and the PAP therapy control PSG parameters was formed for each respective anatomic feature and compared.

### 2.2. Statistics

All data were statistically analyzed using SPSS Statistics Version 23 (IBM Cooperation, Armonk, NY, USA). For categorical variables, we used number and percentage (%). For continuous variables, we used mean ± standard deviation (SD) if normally distributed vs. median and interquartile range (IQR; presented as Q3 minus Q1) if not normally distributed. When patients underwent two full nights of PSG, the mean of both polysomnographic values was calculated. To determine statistical significance when comparing groups based on nasal anatomic features (no nasal obstruction, septal deviation present, turbinate hyperplasia present, septum deviation and turbinate hyperplasia present), an ANOVA test or Kruskal–Wallis test was performed accordingly after assessing the normality of the distribution. The same procedure was applied in a second step for the pharyngeal anatomic features (no pharyngeal obstruction, webbing present, tonsillar hyperplasia present, webbing and tonsillar hyperplasia present). To determine statistical significance when comparing groups based on tongue base anatomy, two groups were formed (no tongue base hyperplasia, tongue base hyperplasia present). After assessing the normality of distribution, a t-test or Mann–Whitney U test was performed accordingly. A *p*-value of <0.05 was considered significant. For graphical illustration, we used column charts in the figures with median and IQR. Graphics were created with GraphPad Prism version 5.01 for Windows. Statistics were performed with advice from the local Institute of Medical Biometry, Epidemiology, and Informatics. 

### 2.3. Ethics

This study was conducted according to the ethical guidelines of the Helsinki Declaration of 1975 (sixth revision, 2008). All included patients had provided informed consent to the use of their data for research. All data were analyzed in an anonymized fashion. All study procedures were in accordance with local data procedures and research practices. The ethics committee of the State Medical Association of Rhineland-Palatinate approved the protocol (2023–17041).

## 3. Results

### 3.1. Baseline Characteristics of the Study Participants

The data of a total of 93 patients were included in this study. Seventy-five (80.6%) patients were male and eighteen (19.4%) patients were female. Age distribution of the study participants was 30–78 years (57.5 ± 13.0 years) and mean BMI was 32.2 ± 5.80 kg/m^2^. The baseline characteristics of the study participants are shown in Table 1. 

### 3.2. Polysomnographic Parameters of the Study Participants

Each patient received full-night PSG in our sleep laboratory for the first time and subsequently began PAP therapy after a diagnosis of OSA. Two full nights of diagnostic PSG were performed in 47 out of 93 patients; this was done to minimize the first night effect of PSG [36]. All parameters examined showed reduced metrics in the PAP therapy control PSG compared with the diagnostic PSG. Table 2 presents polysomnographic parameters of the study population at the time of OSA diagnosis and the results of PAP therapy at the time of PAP therapy control PSG.

### 3.3. Effect of Nasal Obstruction on Positive Airway Pressure Therapy Response

In this initial analysis, we compared polysomnographic parameters before and after the start of PAP therapy in relation to different types of nasal obstruction. In four out of ninety-three patients, no information about nasal features could be found in their medical files; these patients were excluded from the calculations.

The ARI showed no significant differences between all compared groups in the diagnostic or PAP therapy control PSG. The Δ ARI was similar in three out of four groups, but higher in the PAP therapy control group for patients with turbinate hyperplasia (no significance found, *p* = 0.176). Accordingly, the RDI showed no significant differences between all compared groups in the diagnostic or PAP therapy control PSG. Also, the Δ RDI was not significantly different between all compared groups (*p* = 0.475). The AI tended to behave similarly, with no significant differences between groups in the diagnostic or PAP therapy control PSG. The Δ AI was not significantly different between all compared groups (*p* = 0.733). For the HI, a trend toward higher levels was found in patients without nasal obstruction and patients with turbinate hyperplasia, but without statistical significance. The HI was not significantly different between all compared groups in the PAP therapy control PSG. Further, the Δ HI did not differ significantly in any group (*p* = 0.179). Finally, the ODI showed no significant differences between all compared groups in the diagnostic or PAP therapy control PSG. Accordingly, the Δ ODI did not differ significantly in any group (*p* = 0.435). 

**Figure 1:**
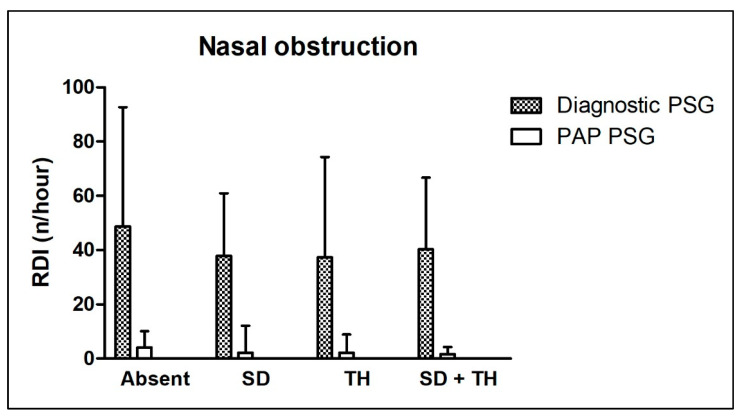
Effect of nasal obstruction on the respiratory disturbance index in diagnostic and positive airway pressure therapy control polysomnography. “Absent”—no nasal obstruction; “SD”—septal deviation present; “TH”—turbinate hyperplasia present; “SD + TH”—septal deviation and turbinate hyperplasia present. Further abbreviations: PAP—positive airway pressure; PSG—polysomnography; RDI—respiratory disturbance index.

Age distribution and BMI did not differ significantly between all compared groups. Table 3 shows detailed information about all the above-mentioned parameters. Figure 1 shows the effect of different types of nasal obstruction on the RDI in the diagnostic and PAP therapy control PSG.

### 3.4. Effect of Pharyngeal Obstruction on Positive Airway Pressure Therapy Response

After investigating sleep parameters in relation to different aspects of anatomic features of the nose, we aimed to further analyze the effect of pharyngeal features on PAP therapy response. In 13 out of 93 patients, no information about pharyngeal features could be found in their medical files; these patients were excluded from the calculations.

In the diagnostic PSG, the ARI was significantly higher in patients with tonsil hyperplasia compared to other groups. The ARI showed no significant differences between all groups in the PAP therapy control PSG. The Δ ARI was significantly higher in the patient group with tonsil hyperplasia (*p* = 0.021). The RDI showed no significant differences between all compared groups in the diagnostic or PAP therapy control PSG. Also, the Δ RDI was not significantly different between all compared groups (*p* = 0.935). The AI tended to behave similary, with no significant differences between groups in the diagnostic or PAP therapy control PSG. The Δ AI was not significantly different between all compared groups (*p* = 0.232). Accordingly, the HI showed no significant differences between all compared groups in the diagnostic or PAP therapy control PSG. Further, the Δ HI did not differ significantly between groups (*p* = 0.462). Finally, the ODI showed no significant differences between all compared groups in the diagnostic or PAP therapy control PSG. Accordingly, the Δ ODI did not differ significantly in any group (*p* = 0.164). 

**Figure 2:**
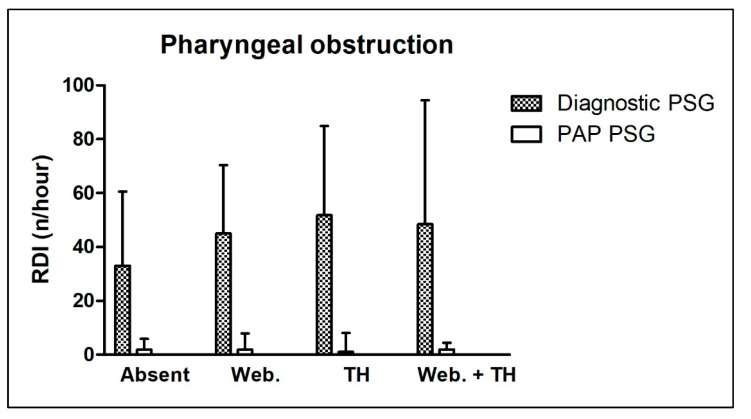
Effect of pharyngeal obstruction on the respiratory disturbance index in diagnostic and positive airway pressure therapy control polysomnography. “Absent”—no pharyngeal obstruction; “Web.”—webbing present; “TH”—tonsil hyperplasia present; “Web. + TH”—webbing and tonsil hyperplasia present. Further abbreviations: PAP—positive airway pressure; PSG—polysomnography; RDI—respiratory disturbance index.

Age distribution and BMI did not differ significantly between all compared groups. Table 4 shows detailed information about all the above-mentioned parameters. Figure 2 shows the effect of different types of pharyngeal obstruction on the RDI in the diagnostic and PAP therapy control PSG.

### 3.5. Effect of Tongue Base Anatomy on Positive Airway Pressure Therapy Response

Finally, we aimed to investigate the effect of tongue base anatomy on PAP therapy response. In seven out of ninety-three patients, no information about tongue base anatomy could be found in their medical files; these patients were excluded from the calculations. 

In the diagnostic PSG, the ARI was higher in patients without tongue base hyperplasia and those with hyperplasia of the tongue base compared to the PAP therapy control PSG. Tongue base anatomy showed no significant difference before or after the start of PAP therapy. The Δ ARI was not significantly different between the compared groups (*p* = 0.744). The RDI showed no significant differences between the compared groups in the diagnostic or PAP therapy control PSG. Also, the Δ RDI was not significantly different between the compared groups (*p* = 0.799). The AI tended to behave similarly, with no significant differences between groups in the diagnostic or PAP therapy control PSG. Also, the Δ AI was not significantly different between the compared groups (*p* = 0.605). In the diagnostic PSG, the HI was significantly higher in patients with hyperplasia of the tongue base compared to those without. Contrarily, there was no significant difference between the compared groups in the PAP therapy control PSG. Also, the Δ HI was not significantly different between the compared groups (*p* = 0.052). Accordingly, the ODI showed no significant differences between the compared groups in the diagnostic or PAP therapy control PSG. The Δ ODI did not differ significantly between the compared groups (*p* = 0.672). 

**Figure 3:**
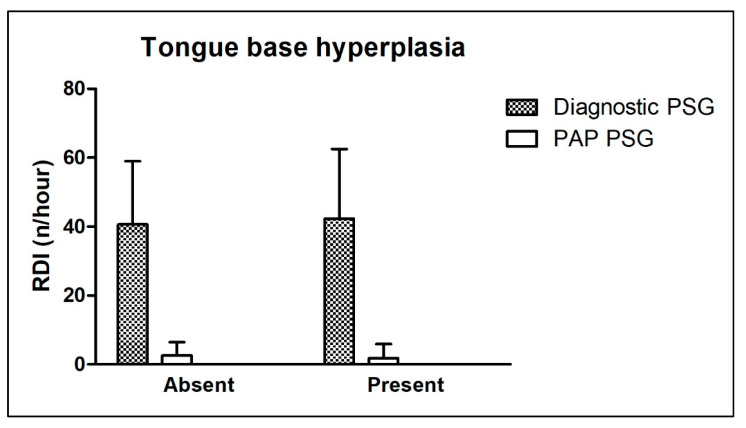
Effect of tongue base anatomy on the respiratory disturbance index in diagnostic and positive airway pressure therapy control polysomnography. “Absent”—no tongue base hyperplasia; “Present”—tongue base hyperplasia present. Further abbreviations: PAP—positive airway pressure; PSG—polysomnography; RDI—respiratory disturbance index.

Age distribution and BMI did not differ significantly between all compared groups. Table 5 shows detailed information about all the above-mentioned parameters. Figure 3 shows the effect of the tongue base anatomy on the RDI in the diagnostic and PAP therapy control PSG.

## 4. Discussion

In this study, we analyzed the effect of nasal and pharyngeal anatomic features on OSA severity and PAP therapy response. In addition to respiratory events as an expression of OSA severity, the arousal index (ARI) and the oxygen desaturation index (ORI) were included in our study. Different anatomic features of nasal obstruction had no significant impact on OSA severity and, more importantly, PAP therapy response. Accordingly, PAP therapy response was not influenced by different anatomic features of pharyngeal obstruction. However, patients with tonsil hyperplasia tended to show a higher ARI in the diagnostic PSG compared to patients without pharyngeal obstruction. Additionally, patients with tongue base hyperplasia showed a significantly higher hypopnea index (HI) in the diagnostic PSG, but tongue base anatomy did not affect the response to PAP therapy. 

Patients with OSA often have abnormalities of the nasal anatomy, and conversely, obstructive changes in the nasal anatomy have been discussed in the literature as a risk factor for the development of OSA [22,23,37,38,39]. However, the pathophysiology of the contribution of nasal obstruction to the severity of OSA is not fully understood. Possible explanations described in the literature include the Starling resistor model, the consecutive switch to less favorable mouth breathing, the nasal reflex, and the absence of nitric oxide. Some studies describe a relationship between nasal obstruction and AHI [22,24], while others find none [25,40]. The assumption that the impact of septal deviation and turbinate hyperplasia on the response to treatment with PAP is small is also supported by the fact that nasal surgery alone is usually unable to adequately treat OSA [41]. However, nasal disease and nasal parameters are considered the main factors for prompt CPAP therapy discontinuation, and pre-CPAP nasal treatment or surgery can be predicted before therapy initiation to guarantee long-term adherence [26]. In fact, excessive upper airway blockage as seen in patients with septal deviation and/or turbinate hyperplasia has been reported to cause subjective discomfort that reduces CPAP compliance [27]. Interestingly, in our cohort, nasal obstruction had no impact on RDI, suggesting that PAP therapy works despite anatomic abnormalities. However, lack of consistent PAP treatment or nonadherence may make it difficult to assess its true efficacy.

In the present study, tonsil hyperplasia and webbing showed no significant negative impact on PAP response. However, ARI, respiratory disturbance index (RDI), and ODI were highest among patients with tonsillar hyperplasia in the diagnostic PSG. These findings are in line with a study by Friedman et al. that determined tonsil hyperplasia to be a predictor of higher RDI [42]. It is likely that tonsillar hyperplasia has a greater impact on the severity of OSA than webbing. However, other authors described no relationship between the severity of OSA and tonsil hyperplasia [28,43]. In addition, Cahali et al. described in their cross-sectional study that only sporadic tonsil hyperplasia grade 4 was suggestive of more severe OSA [29]. In our study, 20% of the patients had tonsil hyperplasia grade 3; this value is slightly higher than described in the literature: Zonato et al. 14.8% [39], Jara et al. 19% [30], Cahali et al. 16.2% [29], and Friedman et al. 9.3% [42]. Furthermore, a tonsillar grade of 4 was described in 3.75% of patients in our study compared with 1.5% in Zonato et al. [39], 5% in Jara et al. [30], 1.5% in Cahali et al. [29], and 3.7% in Friedman et al. [42]. However, the group of those who had only tonsil hyperplasia but no webbing was relatively small (n = 5), so conclusions may have arisen by chance or were biased by the fact that our data were collected from an ears, nose, and throat (ENT) clinic.

In the present investigation, patients with tongue base hyperplasia showed a significantly higher HI in the diagnostic PSG (*p* = 0.025) compared with patients with normal tongue base anatomy. Other PSG parameters did not differ significantly in patients with tongue base hyperplasia compared to those with normal anatomy. This result contrasts with the study by Friedman et al. in which the change in modified Mallampati score (and Friedman tongue position) correlated well with the RDI [42]. A meta-analysis of 10 studies found that the Mallampati classification and Friedman tongue position assessment techniques were significantly correlated with predicting severity of OSA; however, the strength of this correlation was higher for Friedman tongue position [44]. A study by Park et al. compared a group of patients with OSA who regularly used CPAP with a group that did not comply. The authors found that the non-compliant group had a higher incidence of patients with nasal and pharyngeal obstruction. Although nasal or pharyngeal abnormalities have little effect on CPAP response, these results suggested that they do affect CPAP use. In their study, septal deviation and tongue base hyperplasia were associated with lower compliance [27]. In some studies, compliance with CPAP therapy increased after nasal or pharyngeal surgery and the required CPAP pressure was reduced [45,46,47,48]. It is known that obese patients and those with higher grade septal deviation, turbinate hyperplasia, severe tonsillar hyperplasia, or a higher level of palatal grade are more likely to not respond to CPAP therapy [27]. Common complaints of patients who do not adhere to CPAP therapy include subjective complaints such as physical discomfort when wearing the mask, chest discomfort, dry mouth, and nasal obstruction [27]. Therefore, a thorough physical examination of the nasal cavity and oropharynx as well as adequate education about the potential subjective discomfort associated with CPAP treatment is required before prescribing CPAP therapy [27]. Furthermore, it has been discussed that additional therapeutic trials to improve upper airway narrowing prior to initiation of CPAP therapy could improve CPAP compliance [27]. It should be noted here that the results obtained in our study may differ from those of CPAP users because of individual pressure settings, adaptability to changing severity of OSA, and users’ better experience with APAP therapy. In addition, treatment adherence and efficacy may vary among individuals due to other factors, such as the type of mask studied (nasal, facial, etc.). In our study, patients with tongue base hyperplasia showed increased HI. This relationship was already described in a study by Kim et al. and was attributed to increased fat deposition at the base of the tongue [49]. 

Over time, anatomic explanatory models have been supplemented with functional explanatory models, as OSA also occurs in patients with favorable anatomy [6]. Functional explanatory models involve neuromuscular interplay, arousal threshold, and instability of respiratory control, so-called loop gain [6]. In fact, a study by Eckert et al. hypothesized that non-anatomic features play a role in 56% of patients with OSA [3]. 

Because of the cross-sectional design of our study, it was not possible to examine the effect of alterations of the nasal and pharyngeal region on OSA severity and PAP therapy response in each separate study participant. Future studies should address this limitation and more are needed to examine the effects of nasal and pharyngeal obstruction on this subject. An attempt was made to reduce confounding factors such as BMI or age that influence the severity of OSA. However, males constituted the majority of our study sample. This re-occurring sex bias in studies on OSA may limit the population to which the presented results can be applied. Accordingly, this sex bias may have altered the presented results independently. Moreover, the appearance of sleep apnea or PAP response may have been influenced by unreported diseases or past OSA-related surgeries. Further, in the present study, exact grading of hyperplasia was not performed, and a distinction was made only in a binary (present/absent) format, possibly by different examiners. In addition, the examination of the nasal cavity was performed only clinically and not by nasal resistance, computed tomography, or magnetic resonance imaging. This limitation does not allow conclusions to be drawn about the PAP response in more severe forms of nasal obstruction. To address this issue, further studies are needed that consider nasal obstruction not only in a binary format but in an objective manner to assess the exact corresponding PAP response. For example, it is conceivable that an extreme form of nasal obstruction may significantly limit PAP response. Another limitation is that no data on adherence to PAP therapy were available, so conversely, no conclusions could be drawn regarding specific anatomic regions responsible for nonadherence to PAP therapy. Nonadherence to PAP therapy may be due to several reasons; a future study could explicitly address adherence independently based on nasal or pharyngeal anatomic changes. Another limitation is that no long-term data were available to form conclusions. In the present study, part of the patient population was diagnosed by only one full-night PSG. We note this limitation as two full nights of PSG can minimize the so-called first night effect [36].

However, to the best of our knowledge, this is the first study to investigate the extent to which nasal and pharyngeal (velar and tongue base) anatomic features influence PAP therapy response. Most studies on the subject investigated the influence of anatomy on OSA severity, commonly using the AHI. In our work, the RDI, the individual components of AI and HI, and other polysomnographic parameters were included. The following novelty arises for sleep medicine, but its significance should be confirmed in larger collectives: the exact effect of anatomic features of the nasal and pharyngeal region on PAP therapy is vague. Due to interindividual compliance, a detailed otolaryngologic examination and collection of personalized patient information should be performed prior to initiation of PAP therapy. This measure could potentially improve PAP therapy response by creating awareness about the occurrence of potential negative side effects in advance. Therefore, the above issues have additional potential implications for the need and priority of personalized treatment for OSA patients to improve outcomes and quality of life.

## 5. Conclusions

According to this study, anatomic features of the nasal and pharyngeal region have only a minor influence on PAP therapy response. However, anatomic features of the nasal and pharyngeal region should be considered individually to increase therapy compliance in patients with OSA. 

## Figures and Tables

**Table 1:** Baseline characteristics of the study participants.

	Study Participants
Number of patients	93
Number of male patients (%)	75 (80.6)
Number of female patients (%)	18 (19.4)
Age, in years ± SD	57.5 ± 13.0
BMI, in kg/m^2^ ± SD	32.2 ± 5.80

Abbreviation: BMI—body mass index.

**Table 2:** Polysomnographic parameters of the study participants.

	Diagnostic PSG	PAP Therapy Control PSG
ARI (n/hour (IQR))	33.5 (24.4)	17.25 (11.87)
RDI (n/hour (IQR))	40.3 (28.2)	4.20 (4.44)
AI (n/hour (IQR))	36.1 (29.8)	1.38 (3.24)
HI (n/hour (IQR))	5.28 (7.71)	0.30 (1.04)
ODI (n/hour (IQR))	30.4 (33.6)	2.80 (5.96)

Abbreviations (in alphabetical order): AI—apnea index; ARI—arousal index; HI—hypopnea index; IQR—interquartile range; ODI—oxygen desaturation index; PAP—positive airway pressure; PSG—polysomnography; RDI—respiratory disturbance index.

**Table 3:** Baseline characteristics and polysomnographic parameters in relation to nasal anatomic features.

		No Nasal Obstruction	Septal Deviation Present	Turbinate Hyperplasia Present	Septal Deviation and Turbinate Hyperplasia Present	Between-Group Comparison (*p*-Value)
Number of patients (%)		14 (15.7)	18 (20.2)	7 (7.90)	50 (56.2)	
Percentage male		85.7	83.3	42.9	84	
Age, in years (±SD)		60.4 (±10.7)	56.0 (±15.4)	55.9 (±10.2)	57.2 (±13.6)	*p* = 0.796
BMI, in kg/m^2^ (±SD)		30.8 (±4.69)	31.1 (±4.29)	32.8 (±4.41)	32.9 (±6.67)	*p* = 0.512
ARI (n/hour (IQR))	Diagnostic PSG	32.3 (23.3)	34.8 (30.0)	21.7 (25.9)	36.2 (21.3)	*p* = 0.971
	PAP PSG	18.1 (11.3)	21.5 (12.1)	27.1 (16.1)	16.1 (12.5)	*p* = 0.140
RDI (n/hour (IQR))	Diagnostic PSG	48.7 (44.0)	37.9 (23.1)	37.3 (37.0)	40.3 (26.4)	*p* = 0.483
	PAP PSG	4.15 (6.00)	2.15 (9.94)	2.10 (6.75)	1.60 (2.71)	*p* = 0.365
AI (n/hour (IQR))	Diagnostic PSG	43.6 (43.6)	36.6 (30.4)	27.6 (32.7)	36.1 (28.1)	*p* = 0.455
	PAP PSG	2.12 (4.89)	1.92 (9.05)	0.90 (2.55)	1.05 (2.44)	*p* = 0.722
HI (n/hour (IQR))	Diagnostic PSG	6.40 (9.49)	3.23 (5.80)	6.75 (6.10)	3.47 (5.13)	*p* = 0.051
	PAP PSG	0.78 (1.68)	0.43 (1.48)	0.40 (3.25)	0.20 (0.80)	*p* = 0.224
ODI (n/hour (IQR))	Diagnostic PSG	38.1 (36.1)	29.8 (26.0)	24.6 (22.5)	30.7 (31.2)	*p* = 0.439
	PAP PSG	4.83 (6.19)	3.03 (8.06)	2.20 (8.75)	2.30 (3.86)	*p* = 0.387

Abbreviations (in alphabetical order): AI—apnea index; PAP—positive airway pressure; ARI—arousal index; BMI—body mass index; HI—hypopnea index; IQR—interquartile range; ODI—oxygen desaturation index; PSG—polysomnography; RDI—respiratory disturbance index; SD—standard deviation.

**Table 4:** Baseline characteristics and polysomnographic parameters in relation to pharyngeal anatomic features.

		No Pharyngeal Obstruction	Webbing Present	Tonsil Hyperplasia Present	Webbing and Tonsil Hyperplasia Present	Between-Group Comparison (*p*-Value)
Number of patients (%)		23 (28.8)	38 (47.5)	5 (6.30)	14 (17.5)	
Percentage male		69.6	81.6	100	78.6	
Age, in years (±SD)		58.5 (±12.7)	59.3 (±12.6)	49.0 (±11.3)	50.4 (±12.8)	*p* = 0.067
BMI, in kg/m^2^ (±SD)		31.3 (±6.53)	32.0 (±5.34)	33.4 (±1.30)	34.61 (±7.30)	*p* = 0.387
ARI (n/hour (IQR))	Diagnostic PSG	30.0 (18.1)	34.1 (24.44)	59.0 (22.8)	42.4 (26.7)	*p* = 0.018
	PAP PSG	18.1 (16.3)	16.2 (13.1)	24.4 (29.8)	20.2 (5.34)	*p* = 0.164
RDI (n/hour (IQR))	Diagnostic PSG	33.0 (27.5)	45.0 (25.4)	51.8 (33.1)	48.5 (46.0)	*p* = 0.131
	PAP PSG	1.90 (4.00)	1.85 (6.00)	1.00 (7.08)	1.92 (2.50)	*p* = 0.859
AI (n/hour (IQR))	Diagnostic PSG	27.6 (28.3)	38.8 (30.7)	35.6 (31.3)	39.4 (44.0)	*p* = 0.176
	PAP PSG	1.70 (3.30)	1.20 (4.50)	0.70 (5.30)	1.60 (2.00)	*p* = 0.935
HI (n/hour (IQR))	Diagnostic PSG	5.80 (7.35)	5.40 (8.90)	3.00 (7.24)	3.73 (46.0)	*p* = 0.468
	PAP PSG	0.00 (0.90)	0.55 (1.55)	0.10 (0.70)	0.25 (0.71)	*p* = 0.107
ODI (n/hour (IQR))	Diagnostic PSG	22.6 (20.45)	33.8 (30.3)	45.2 (49.9)	31.9 (62.1)	*p* = 0.165
	PAP PSG	2.70 (4.30)	3.15 (8.00)	2.95 (7.95)	2.95 (3.43)	*p* = 0.811

Abbreviations (in alphabetical order): AI—apnea index; PAP—positive airway pressure; ARI—arousal index; BMI—body mass index; HI—hypopnea index; IQR—interquartile range; ODI—oxygen desaturation index; PSG—polysomnography; RDI—respiratory disturbance index; SD—standard deviation. *p*-values in bold are statistically significant.

**Table 5:** Baseline characteristics and polysomnographic parameters in relation to tongue base anatomy.

		No Tongue Base Hyperplasia	Tongue Base Hyperplasia Present	Between-Group Comparison (*p*-Value)
Number of patients (%)		33 (38.8)	53 (61.2)	
Percentage male		87.9	75.0	
Age, in years (±SD)		57.7 (±15.3)	56.8 (±11.6)	*p* = 0.749
BMI, in kg/m^2^ (±SD)		32.62 (±7.43)	31.93 (±4.72)	*p* = 0.632
ARI (n/hour (IQR))	Diagnostic PSG	33.2 (24.9)	33.7 (24.0)	*p* = 0.595
	PAP PSG	16.6 (17.5)	18.2 (11.2)	*p* = 0.847
RDI (n/hour (IQR))	Diagnostic PSG	40.6 (18.4)	42.3 (20.2)	*p* = 0.701
	PAP PSG	2.60 (3.83)	1.75 (4.13)	*p* = 0.691
AI (n/hour (IQR))	Diagnostic PSG	35.3 (20.4)	33.4 (20.0)	*p* = 0.662
	PAP PSG	1.4 (2.95)	1.25 (2.85)	*p* = 0.755
HI (n/hour (IQR))	Diagnostic PSG	3.45 (5.40)	5.82 (10.3)	*p* = 0.025
	PAP PSG	0.95 (1.13)	0.30 (0.88)	*p* = 0.843
ODI (n/hour (IQR))	Diagnostic PSG	30.8 (31.3)	30.0 (31.2)	*p* = 0.709
	PAP PSG	2.95 (3.80)	2.10 (6.88)	*p* = 0.850

Abbreviations (in alphabetical order): AI—apnea index; PAP—positive airway pressure; ARI—arousal index; BMI—body mass index; HI—hypopnea index; IQR—interquartile range; ODI—oxygen desaturation index; PSG—polysomnography; RDI—respiratory disturbance index; SD—standard deviation. *p*-values in bold are statistically significant.

## Data Availability

The data presented and analyzed in this study are available on reasonable request from the corresponding author.
